# The New York Drug-Clerk Law

**Published:** 1871-06

**Authors:** 


### The New York Drug-Clerk Law.

"SECTION 1. The Mayor of the city of New York is hereby authorized to appoint, within ninety days after the passage of this act a board, to consist of one skilled pharmaceutist, one practical druggist, and two regular physicians of the city of New York, to hold office during the pleasure of said Mayor, to act as an examining board for the examination and licensing of all druggists and persons now employed, or hereafter to be employed, as clerks by any druggist, keeper, proprietor, or superintendent of any drug store in said city, who shall be engaged in preparing and putting up physicians' prescriptions or dispensing medicine. On and after six months from the date of the organization of such board, any person who shall not have passed an examination before and received a certificate from said board, who shall make up, or attempt to make up, a prescription—any physicians' prescription—shall be deemed guilty of a misdemeanor, and shall, upon conviction thereof, be fined not more than $500, or imprisoned not longer than six months, or both, at the discretion of the Court.

"SEC. 4. It shall be the duty of said board to examine, on application, all persons employed, or hereafter to be employed, in putting up prescriptions or dispensing medicine in the city of New York, and give a certificate of such exanination to the person so examined, if found competent to act in such capacity, and which certificate shall be deemed as a license for such person to engage in such employment.

"SEC. 5. Said board shall, with the approval of the Mayor, fix the sum to be paid for such certificates by the persons to whom they shall be issued, and all sums or fees for certificates raised by said board shall be appropriated to the payment of the expenses and salaries of the members of said board, or so much thereof as may be necessary, the balance, if any, to be paid into the city treasury. Said board shall cause a true and accurate account of its receipts and disbursements to be kept, and shall, once in three months make a return of the amounts received and expended to the Controller of the city of New York."

